# A microfluidic paper analytical device using capture aptamers for the detection of *Pf*LDH in blood matrices

**DOI:** 10.1186/s12936-022-04187-6

**Published:** 2022-06-07

**Authors:** Adewoyin Martin Ogunmolasuyi, Ronen Fogel, Heinrich Hoppe, Dean Goldring, Janice Limson

**Affiliations:** 1 Biotechnology Innovation Centre, Rhodes University, P.O. Box 94, Grahamstown, 6140 Eastern Cape South Africa; 2 Department of Biochemistry and Microbiology, Rhodes University, P.O. Box 94, Grahamstown, 6140 Eastern Cape South Africa; 3 Department of Biochemistry, University of KwaZulu-Natal, Private Bag X01, Scottsville, Pietermaritzburg, 3209 KwaZulu-Natal South Africa

**Keywords:** Malaria, Paper, Diagnostics, Aptamer, *Plasmodium falciparum* LDH test, Blood

## Abstract

**Background:**

The prevalence and death rate arising from malaria infection, and emergence of other diseases showing similar symptoms to malaria require the development of malaria-specific and sensitive devices for its diagnosis. To address this, the design and fabrication of low-cost, rapid, paper-based analytical devices (µPAD) using surface-immobilized aptamers to detect the presence of a recombinant malarial biomarker—*Plasmodium falciparum* lactate dehydrogenase (r*Pf*LDH)—is reported in this study.

**Methods:**

Test zones on paper surfaces were created by covalently immobilizing streptavidin to the paper, subsequently attaching biotinylated aptamers to streptavidin. Aptamers selectively bound r*Pf*LDH. The measurement of captured r*Pf*LDH enzyme activity served as the means of detecting this biomarker. Enzyme activity across three replicate sensors was digitally quantified using the colorimetric Malstat assay.

**Results:**

Screening of several different aptamers reported in the literature showed that aptamers rLDH7 and 2008s immobilized in this manner specifically recognised and captured *Pf*LDH. Using rLDH7, the sensitivity of the µPAD sensor was evaluated and the µPAD sensor was applied for preferential detection of r*Pf*LDH, both in buffered solutions of the protein and in spiked serum and red blood cell lysate samples. In buffered solutions, the test zone of the µPAD sensor exhibited a *K*_*D*_ of 24 ± 11 nM and an empirical limit of detection of 17 nM, respectively, a limit similar to commercial antibody-based sensors exposed to r*Pf*LDH. The specific recognition of 133 nM r*Pf*LDH in undiluted serum and blood samples was demonstrated by the µPAD.

**Conclusion:**

The reported µPAD demonstrates the potential of integrating aptamers into paper-based malarial rapid diagnostic tests.

**Graphical Abstract:**

**Figure:**
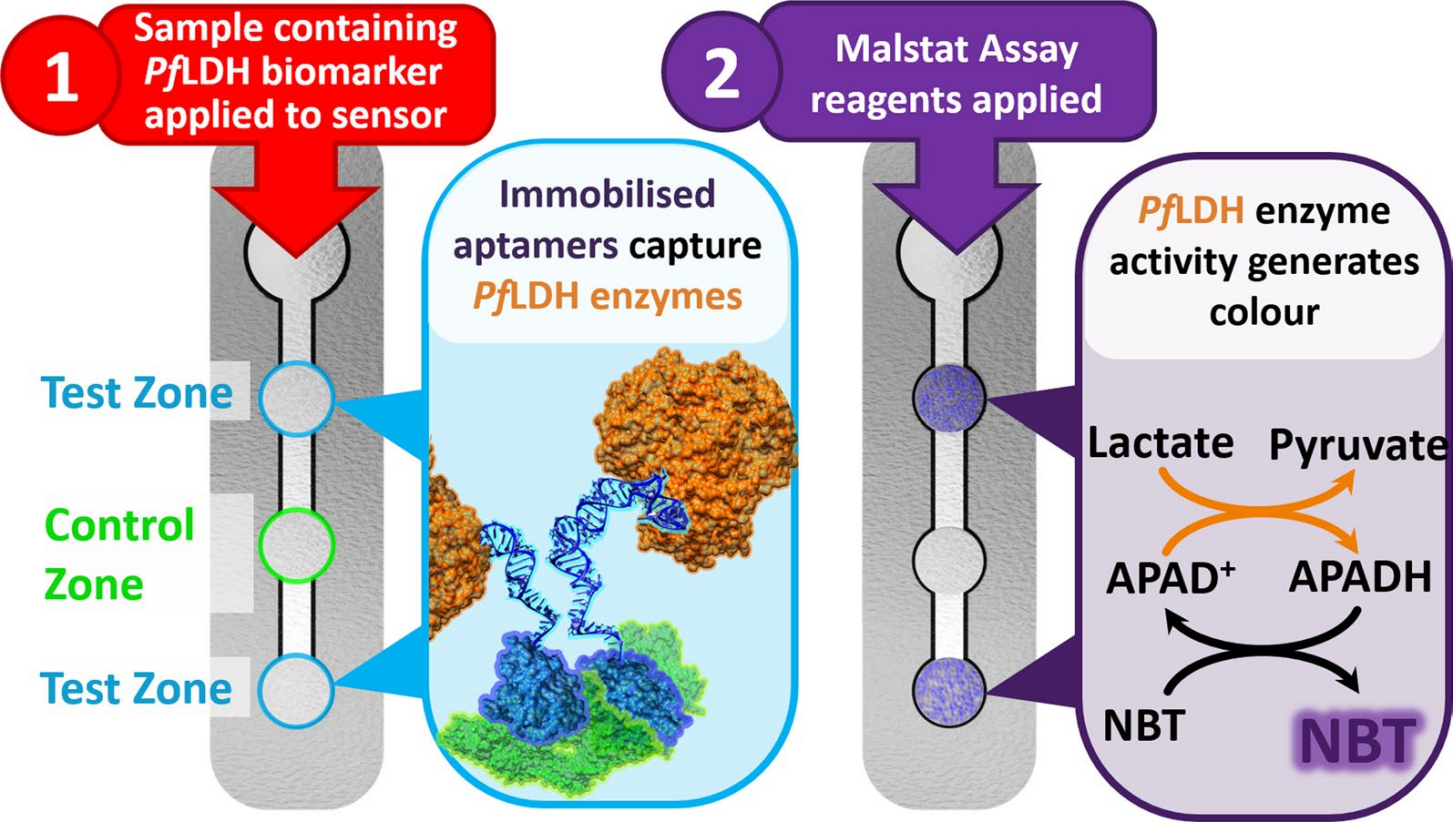
The assembly of µPAD sensors using APTEC assay principles for the detection the malarial biomarker, lactate dehydrogenase enzymes from Plasmodium falciparum (PfLDH). The aptamers immobilized at the test zones capture the PfLDH in samples. After washing the unbound sample components from the zones, Malstat assay reagents are added for colour development, proportional to the amount of captured PfLDH.

**Supplementary Information:**

The online version contains supplementary material available at 10.1186/s12936-022-04187-6.

## Background

The continued mortality and morbidity caused by the malarial parasite *Plasmodium falciparum* in certain African regions, where over 90% of all malaria cases occurs, was the basis of the distribution of 412 million rapid diagnostic tests (RDT) for malaria in 2018 [1]. Tests that can offer a species-specific approach in the diagnosis of malaria can help guide decision-making in effective treatment [2]; responding to this, over 64% of malaria tests distributed in 2018 were specific to *P. falciparum* [3]. The majority of rapid diagnostic tests (RDTs) used in the field to diagnose malaria infections detect PfHRP2, a histidine-rich protein which is only expressed by *P. falciparum* parasites [1]. While still an active area of research e.g. Lo et al. [4], there are increasing numbers of isolates of *P. falciparum* from different regions that lack the *pfhrp2* gene and hence RDTs detecting PfHRP2 no longer detect many *P. falciparum* infections [5]. There is, therefore, scope in developing new malaria detection methods to supplement the existing PfHRP2 RDTs. The lactate dehydrogenase enzyme expressed by *Plasmodium* species (*P*LDH) is frequently used as diagnostic biomarkers of malaria [6, 7]. *P*LDH has several favourable features as a biomarker: high expression rates by *Plasmodium* cells [7], multiple structural and kinetic differences between these enzymes and their human counterparts [7], and the variation in amino acid sequence between different *Plasmodium* species [8], which permits identification of the precise species of malaria within a sample. Antibodies against *P*LDH have been shown to differentiate between species [9].

Despite its advantage as a biomarker, commercial rapid diagnostic tests for malaria based on detection of *P*LDH that distinguishes between different species of *Plasmodium* remains limited [1]. Currently, most available rapid diagnostic tests rely on lateral flow assay systems, where the flow of fluid sample through the sensor strip allows the target to be carried to various zones on the strip where labelling and/or capturing of the labelled target takes place via biorecognition [3]. For most lateral flow assays, antibodies which interact specifically with the biomarker to generate a signal e.g. gold nanoparticle aggregation are the preferred biorecognition agent [3]. Recent studies have explored the use of DNA-based aptamers as biorecognition agents in malaria diagnostics. Several aptamers capable of binding to (*P*LDH) [10–12], have been reported (Table 1).

**Table 1:** Summary of aptamers reported previously to bind to *P*LDHs that were used in this study

Aptamer	Reported sequence, sourced for this study (5′-3′)	Binding buffer used	Reference
LDHp1	**GCCTGTTGTGAGCCTCCTAAC**CAGGAAGCGACCTACTAAAGTGATATTAT AGATTCACGGGAGCGTGGTG**CATGCTTATTCTTGTCTCCC **	2 mM HEPES, 0.2 mM MgCl_2_, 0.2 mM CaCl_2_, 0.2 mM KCl, 15 mM NaCl, pH 7.4	[12]
LDHp11	**GCCTGTTGTGAGCCTCCTAAC**CTACTGTTGATATGAGTGATAGGGCGGCG CGCTTATCTAGTGTATTGTG**CATGCTTATTCTTGTCTCCC **		
rLDH4	**GCCTGTTGTGAGCCTCCTAAC**CAGCTCGTAGAAAAAAAAAGATATTGCTTCAATTATCTCCTCGCGTTCAATTAACCCAG**CATGCTTATTCTTGTCTCCC**		
rLDH7	**GCCTGTTGTGAGCCTCCTAAC**CCAGAATAGGGACTGCTCGGGATTGCGGA TGAGTCTGGGTGGGACATGG**CATGCTTATTCTTGTCTCCC**		
rLDH15	**GCCTGTTGTGAGCCTCCTAAC**TTTAAAGTTGCTATTTAACCAAAAAAAAAAAAATAAAAAAGTCGAGCCGGCC**CATGCTTATTCTTGTCTCCC**		
pL1	**CACCTAATACGACTCACTA**TAGCGGATCCGACTCACGTACAGCAAGGTTC GATTGGATTGTGCCGGAAGTGCTGGCTCGA**ACAAGCTTGC**	20 mM Tris–HCl, 50 mM NaCl, 5 mM KCl, 5 mM MgCl_2_, 300 mM imidazole, pH 8.0	[10]
2008s	**CGTACGGTCGACGCTAGC**CTGGGCGGTAGAACCATAGTGACCCAGCCGTC TAC**CACGTGGAGCTCGGATCC**	10 mM Na_2_HPO_4_, 1.8 mM KH_2_HPO_4_, 2.7 mM KCl, 137 mM NaCl, pH 7.4	[11]

Bolded sections of the reported sequences refer to primer-binding sites used during SELEX, while unbolded sequences refer to the variable regions.

The inherent enzyme activity of *P*LDH can be used to generate a specific colorimetric signal for diagnosis of malaria infection [6]; similarly, *P*LDH activity in infected erythrocytes is used to evaluate new antimalarial drugs [13]. In the Malstat test, *P*LDH in a sample utilizes the cofactor 3-acetylpyridine adenine dinucleotide (APAD^+^)—an analogue of nicotinamide adenine dinucleotide (NAD^+^)—to oxidize L-lactate to form pyruvate [14]. The reduced form of APAD^+^, APADH, is then subsequently used to reduce tetrazolium dyes to generate a colorimetric signal [2, 6].

During the Malstat assay, nonspecific interference from blood samples is minimized somewhat through the creation of conditions that favour *P*LDH enzyme activity over human LDH enzymes: the use of APAD^+^ as an enzyme cofactor [6, 14]; the higher catalytic activity that *P*LDH possesses towards lactate [14]; and the use of elevated lactate concentrations during testing to exploit the lower levels of substrate inhibition found in *P*LDH [15]. Despite these, Malstat tests remain difficult to quantify in red blood cell lysates samples, due to the inherent colour of this matrix and other interferents [6].

To circumvent this limitation, both antibodies e.g. [16, 17] and aptamers e.g. [2, 18, 19] have been immobilized onto solid supports to create biorecognition elements. These have been subsequently used to separate *Pf*LDH from samples and to concentrate it prior to Malstat assaying, which enhances the specificity and sensitivity of detection of this protein. Using this approach, both 2008s and pL1 aptamers were applied to the detection of *Pf*LDH within blood samples [2, 18], 2008s being further integrated into a microfluidic analytical device [19].

Significant scope exists to develop aptamer-based devices for malaria diagnostics due to their lower production costs, compared to antibodies. Microfluidic paper analytical devices (µPADs), represent a low-cost biosensor technology that is applicable to the detection of *Pf*LDH. µPADs combine the inherent benefits of sample pretreatment and easy-to-understand signal generation of colorimetric paper-based diagnostics[20–23]. µPADs can also include detailed microfluidic structures printed on them using hydrophobic materials, to confine and direct the flow of liquids [24]. Solid ink printing is possibly the most cost-effective method: printing the outlines of the channels onto the paper surface using solid ink printing [25–27] and subsequent thermal treatment to allow the solid ink to permeate through the paper to create a channel.

Building on the advantages of µPAD-based detection (separation of blood component and reduction of nonspecific binding), this study reports on the fabrication of a simple paper microfluidic device for the detection of *Pf*LDH in blood, screening five aptamer sequences reported to bind to *Pf*LDH. Biotinylated rLDH7 was integrated into µPADs as a capture aptamer for *Pf*LDH and served as proof-of-concept for the fabrication of a paper-based microfluidic lateral flow aptasensor for malaria detection.

## Methods

### Reagents and apparatus

The purity of all reagents used in this study were of analytical grade (≥ 95%) or higher, unless otherwise stated. All water used in this study was purified using Millipore’s Direct-Q^®^ water purification system and was of double-distilled (≥ 18.2 MΩ.cm) quality.

The following reagents were sourced from Sigma-Aldrich: Methyl Ester Sulfonic acid (MES), 1-ethyl3-(3-dimethylaminopropyl) carbodiimide hydrochloride (EDC), N-hydroxy-succinimide ester (NHS), Tween^®^20, sodium L-lactate, Triton X-100 (CAS: 9002-93-1), Tris(hydroxymethyl)aminomethane, 3-acetylpyridine adenine dinucleotide (APAD^+^), phenazine ethosulphate (PES), potassium dibasic phosphate, disodium monobasic phosphate, potassium chloride and sodium chloride. p-Nitro blue tetrazolium (NBT) was sourced from Invitrogen.

Phosphate-buffered saline (PBS), pH 7.4 was used to dilute the aptamers prior to paper surface modification, as well as serving as the binding buffer for LDHp11, rLDH4, rLDH7 [12], and 2008s aptamers [11] (Table 1). This was prepared using 10 mM Na_2_HPO_4_, 1.8 mM KH_2_HPO_4_, 2.7 mM KCl, and 137 mM NaCl. The binding buffer used for pL1 aptamer studies was a Tris-based buffer, formulated using 50 mM NaCl, 5 mM KCl, 5 mM MgCl_2_, 0.01% ^v^/_v_ Tween 20 and 20 mM Trizma^®^ base, adjusted with HCl to a pH of 8.0 (Table 1) [10]. Phosphate-buffered saline containing Tween^®^-20 (PBS-T) was formulated using 10 mM Na_2_HPO_4_, 1.8 mM KH_2_HPO_4_, 2.7 mM KCl, 37 mM NaCl, and 0.05% ^v^/_v_ Tween^®^-20 was employed as a washing buffer for all sensors. Whatman chromatography paper, 1CHR, 200 mm x 200 mm, CAT No 3001–861 (GE Healthcare Life Sciences, NY, USA) was employed for the manufacture of the µPAD.

Recombinant lactate dehydrogenase from *P. falciparum* (r*Pf*LDH) was expressed in *Escherichia coli* host cells and purified to a concentration of 2.5 mg.ml^−1^, according to [8]; r*Pf*LDH protein was formulated in 4000 ng.ml^−1^ stocks, equivalent to 133.3 nM. Bovine serum albumin (BSA) was sourced from Biowest and formulated as required in PBS. Streptavidin was isolated from *Streptomyces avidinii* (Catalogue number: S0677) was purchased from Sigma-Aldrich and formulated as 1 mg/ml stock solutions in PBS. Aptamers were diluted from stocks in the binding buffer required for each of the tested aptamers at the required concentrations.

The sequences of the sourced aptamers used in this study (LDHp11, rLDH4, rLDH7, pL1 and 2008s) are detailed in Table 1. All aptamers were biotinylated at the 5′ end and HPLC-purified (Integrated DNA Technologies). All sourced aptamers were prepared as 100 μM stock solutions in Tris–EDTA buffer (10 mM Tris, 1 mM EDTA, pH 8.0) and stored at − 20 °C until used. When required, all aptamers were diluted in their binding buffer to a concentration of 2 μM, heated to 95 °C for 5 min and cooled to room temperature before use. Commercial, antibody-based *Pf*LDH-detecting RDTs (“OnSite^®^ Malaria Pf/Pan Ag Rapid tests”) were sourced from CTK Biotech, Inc. RDTs were operated as per manufacturer’s instructions.

The Malstat assay reagent was prepared similarly to other reports [2, 6, 28]: APAD^+^ (final concentration 0.664 mM) was added to 40 ml of MilliQ water, and dissolved by adjusting the pH to 9.0 using 1 M NaOH. To the APAD solution, sodium L-lactate (final concentration of 0.71 M) and Tris base (0.22 M), TritonX-100 (0.2%) were added and the solution were diluted to 50 ml with water. Separately, 2.5 mM of NBT and 0.299 mM of PES were dissolved in 50 ml of MiliQ water, which was protected from light and stored at 4 °C until used [28].

## Methodology

### Fabrication of microfluidic paper analytical device (µPAD)

**Fig. 1:**
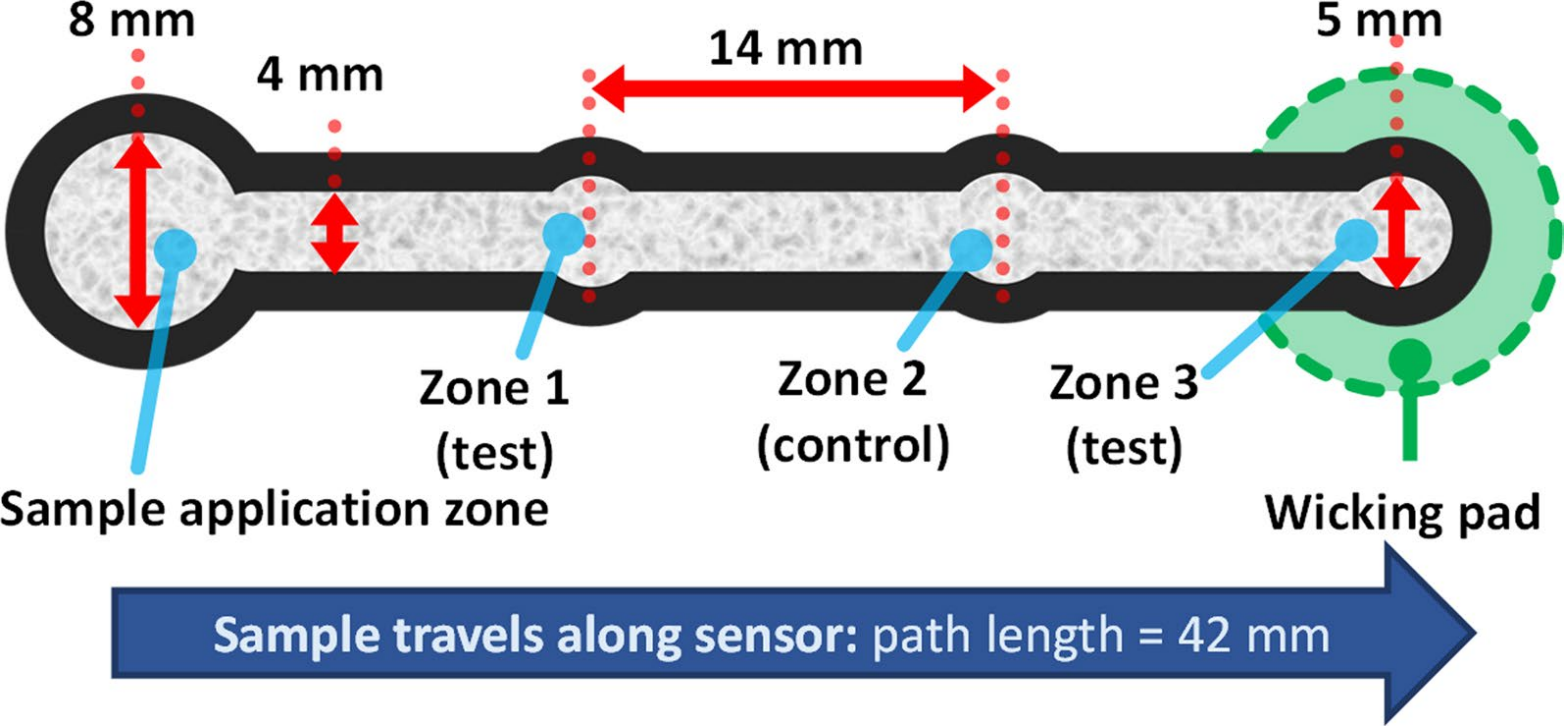
Design of the microfluidic paper analytical device (µPAD) aptasensor used in this study, with relevant dimensions annotated. The flow of fluid is from left-to-right in the above figure. The sample reservoir is an 8 mm-wide circle. Zones 1, 2 and **3**(circles, 5 mm in diameter) are areas where aptamers can be immobilised. Zones are joined together by a 4 mm-wide channel

A microfluidic paper analytical device (µPAD) (Fig. 1) was designed using CorelDRAW software and printed on Whatman chromatography paper using a thermal transfer solid-ink printer (Xerox Colorqube 8870), based at the Council for Scientific and Industrial Research (CSIR) Microfluidic Facility, South Africa.

All fluids, including the blocking agent (BSA), washing buffer, sample (r*Pf*LDH), Malstat reagent and NBT/PES were applied to the sample reservoir using a micropipette and flowed through the channel created by the hydrophobic barrier. At Zone 3, liquid exits the sensor via a wicking pad placed on the underside of Zone 3.

Several zones were designed for the µPAD sensor (Fig. 1). The sample reservoir was about 8 mm in diameter (twice the channel width) to allow for complete absorption of the fluid into the sensor paper before the capillary movement was initiated. The flow path of the paper microfluidic device consisted of three circular zones (5 mm diameter), joined by a 4 mm-wide channel (Fig. 1). Aptamers were immobilized on two of the zones (Zones 1 and 3) for subsequent capture and detection of the target analyte, leaving Zone 2 of each µPAD as a negative control to determine the extent of aptamer-mediated r*Pf*LDH capture.

The total length of the printed paper microfluidic device was 50 mm. The device was bordered with a hydrophobic barrier of 2.12 mm thickness, created using wax ink to print the outline of the µPAD. After printing the design on the chromatography paper using a solid ink printer the paper was heated on a hot plate to 175 °C for 50 s in order to melt the solid ink into the paper, creating a three-dimensional hydrophobic barrier.

Application of reagents and samples was performed on the front (solid ink-printed) side. Apart from the wicking pad under Zone 3, the paper sensor was placed on a polycarbonate plastic sheet, to prevent liquid exiting the sensor by blotting onto a porous surface.

### Construction of the APTEC µPAD

The immobilization of biotinylated aptamers LDHp11, rLDH4, rLDH7, pL1 or 2008s (Table 1) was performed through streptavidin–biotin interactions at Zones 1 and 3 of the µPAD (Fig. 1). Briefly, 2.5 μL of 400 mM EDC and 100 mM NHS solution (in 100 mM MES buffer, adjusted to pH 5.5 with 0.5 M NaOH) was added to the surface of Zones 1 and 3 (Fig. 1) to activate the carboxylic acid groups [29, 30]. Activation proceeded for 1 h at 4 °C, and was repeated by the addition of fresh EDC/NHS solution and re-incubation for another hour. To remove unbound EDC and NHS, three 10 μL aliquots of MES buffer (pH 5.5) was added to the sample area and allowed to flow across the entire µPAD at 10-min intervals.

Following EDC/NHS activation, 1 µl of a 100 µg/ml solution of streptavidin in PBS was aliquoted onto the activated zones on the paper surface and incubated for 2 h at room temperature. The paper device was washed 3 times by addition of 30 µl of PBS-T (pH 7.4) to remove any physically-adsorbed streptavidin from the surface, and the paper was allowed to dry for 5 min, by placing it (facing upwards) onto the surface of another, dry, filter paper. To block residual succinimidyl residues resulting from EDC/NHS activation, 5 µl aliquots of 3% ^w^/_v_ BSA were added to each streptavidin-containing zone and incubated for 30 min at room temperature [29]. Thereafter, the entire sensor surface was blocked by applying 30 µl of 3% ^w^/_v_ BSA across the sensor and incubating it for 30 min at room temperature [29]. The wicking pad was then applied to the underside of Zone 3 of the sensor to remove excess liquid. Unbound BSA was removed by adding 3 × 10 μl PBS-T washing buffer [31] to the sample application zone and wicking excess fluid at Zone 3. Following washing, excess fluid was drained by placing the device onto dry filter paper-based wicking pads after each of the additions of washing buffer.

Aptamers were immobilized to Zones 1 and 3 by adding 1 µl of 2 µM biotinylated aptamer to each zone; allowing the aptamers to bind to the immobilized streptavidin for 1 h at room temperature. Subsequently, the µPAD was washed with PBS-T, exchanging wicking pads between washes. The sensor was used immediately after drying on filter paper until dry (approximately 15 min at room temperature).

### APTEC µPAD colorimetric assay for capture of r*Pf*LDH

A 30 µl aliquot of binding buffer for each of the aptamers was dropped on the sample area of fabricated µPADs and allowed to flow across the channel path (~ 10 min). Following this, 30 µl of sample solution containing r*Pf*LDH was allowed to flow horizontally over the µPAD for 30 min at room temperature to allow aptamer-target interaction. The paper surface was thereafter washed with binding buffer (3 × 10 μL) to remove weakly-bound r*Pf*LDH and dried using a filter paper backing as wicking pad at ambient temperature for about 15 min.

After aptamer-r*Pf*LDH interaction, 30 µl of Malstat reagent and 30 µl NBT/PES solution were mixed together and added to the sample reservoir. These were allowed to flow across the channel path and incubated for 30 min at room temperature to allow colour development. The entire sensor was dried by resting the sensor on paper towelling before capturing a digital image for further analysis.

### Analysis of colorimetric signal

**Fig. 2:**
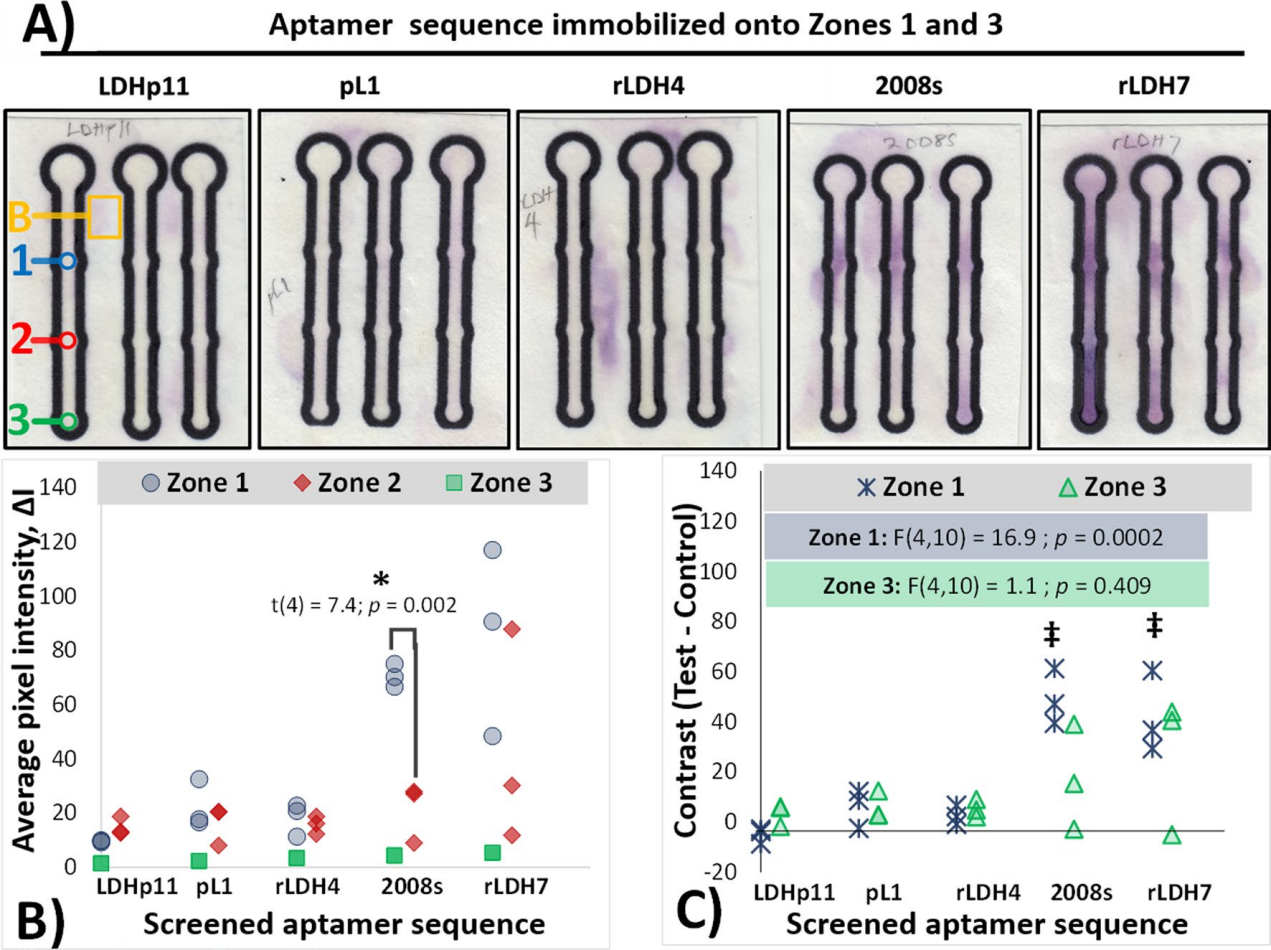
Screening of aptamers for ability to capture 133 nM of r*Pf*LDH for the development of APTEC-based µPAD biosensors. **A** Enhanced-colour scanned images of the tested µPAD sensors following exposure to *rPf*LDH and subsequent Malstat staining of captured enzyme. LDHp11, rLDH4, rLDH7, pL1 and 2008s aptamers were screened in this study. The original scanned images are presented in Additional file information (Additional file 1: Fig. S2). Unenhanced images were used to construct the measurements of *∆I* presented in Fig. 2B nd C. Figure legend (annotations at the left) show the various zones monitored for the measurement B – background area of the test; 1 –Zone 1 (test zone); 2 –Zone 2 (control zone); 3 – Zone 3 (test zone and wicking area). **B** Comparison of the analysed colorimetric intensity of the aptamer-r*Pf*LDH complex after colour development, *∆I* vs. the background. *- indicates screened aptamer responses with Zones 1 and 3 exhibiting significant difference in measured colorimetric intensities compared to its Zone 2 control (*p* ≤ 0.025; two-tailed, unpaired, Student’s t-test). **C **Comparison of the contrast of individual sensors (the difference in colour intensity between the test zones and the control zones for individual sensors). Annotation shows results of the comparison of ANOVA analysis comparing the influence of the sequence tested with the mean contrast obtained at µPAD sensors. ‡—indicates significant difference in a particular aptamer’s colorimetric intensity for zones 1 and 3, compared to those obtained using LDHp11 aptamer (Tukey post hoc test, *p* ≤ 0.05)

The images of the colorimetric signal of the assays were captured by scanning the µPAD using a flatbed scanner (CanoScan LiDE 110). Digital image files (600 dpi Bitmap images) of the µPAD sensors served as the basis for all quantitative analyses. Digital images were analysed using ImageJ software for analysis of the colour intensities [32]. The colour intensity values were obtained by selecting the region of interest and measuring the average RGB intensity of the region using the “RGB Measure” plugin. For each sensor, Zones 1, 2, and 3 (Fig. 1) were measured. Additionally, a region of the paper surface next to each µPAD—outside of the printed confines of the sensor—was measured as the background measurement. Figure 2A below shows examples of these regions sampled for colour determination as annotations.

For each of the zones in the sensors, the colorimetric intensity, ∆*I*, produced by the sensor was calculated using Eq. 1. ∆*I* was set as the magnitude of the vector between the background RGB signal (R_0_, G_0_, B_0_) and the RGB value of the sample (R_n_, G_n_, B_n_):

1 $$\Delta I = \sqrt {\left( {R_{n} - R_{0} } \right)^{2} + \left( {G_{n} - G_{0} } \right)^{2} + \left( {B_{n} - B_{0} } \right)^{2} }$$

where (*R*_0_;_0_;*B*_0_) are the average intensities of the red, green and blue channels measured for the background region for each sensor and (*R*_*n*_;*G*_*n*_;*B*_*n*_) are the average RGB measurements of the zone [32].

The colorimetric responses of test zones of individual sensors were then determined as contrast from the control zones (Eq. 2):

2 $$Contrast = \Delta I_{Test} - \Delta I_{Control}$$

where ∆*I*_*Test*_ is the *ΔI* measurement associated with a test zone for a particular sensor (as calculated in Eq. 1) and ∆*I*_*Control*_ is the colorimetric intensity of the designated control zone in each sensor, Zone 2.

Strictly for presentation purposes in the figures of this study, colour-enhancement of the digital images of the sensors was conducted to aid visibility of produced colour to the reader. This was conducted for all presented images by using the Curves Tool in GNU Image Manipulation Program v2.10.2 (https://www.gimp.org/downloads/), decreasing input RGB values of 85 to an output of 15 and subsequently allowing a smooth curve to adjust all other RGB values in the image. The original, unaltered, images of the sensor were used for calculation of *∆I*; these images are presented in the Additional file Materials.

### Sensitivity and affinity analyses of rLDH7-based APTEC µPADs

The affinity of the fabricated APTEC µPADs to varying concentrations of r*Pf*LDH was prepared by testing the responses of fresh rlDH7-based APTEC µPADs to a concentration range of 0, 1.82, 3.65, 7.30, 14.60, 29.20, 58.40, and 116.80 nM of r*Pf*LDH. 30 µl aliquots of each concentration were tested and analysed as described above. Using these responses, the affinity constants of the APTEC µPAD were fitted to a Langmuir binding isotherm, Eq. 3 [33]:

3 $$\Delta I = \frac{{I_{\max } \times \left[ {\,target} \right]}}{{K_{D} + \left[ {target} \right]}} + I_{0}$$

where *[target]* is the concentration of the target protein used for the paper-based assay in nM, Δ*I* is the change in the colorimetric intensity obtained at a µPAD sensor at a given concentration of target (calculated as in Eq. 1) and ${I}_{0}$ is the baseline response. The apparent dissociation constant of the aptamer-r*Pf*LDH complex, *K*_*D*_ (nM), and the extrapolated maximum colorimetric intensity of the aptamer-target complex, *I*_*max*_*,* were estimated from this model.

The limit of detection was determined empirically, as the lowest tested concentration of *rPf*LDH capable of producing significant sensor contrast i.e. producing an average sensor contrast significantly above zero.

### Detection of r*Pf*LDH in blood and serum matrices

Human serum and red blood cell lysate samples were sourced from the Center for Chemico- and Biomedicinal Research, (Rhodes University). These were obtained in accordance with the ethics application 2011Q4-1, as approved by the Rhodes University Ethical Standards Committee.

Red blood cell samples were lysed by incubating a 50 µL aliquot of whole blood with 100 μL of RBC lysis buffer (155 mM NH_4_Cl, 12 mM NaHCO_3_, and 0.1 mM EDTA) at room temperature under mild agitation for 10 min [10]. Blood lysates were serially diluted with PBS buffer to various haematocrit levels: from 33.3% (i.e. undiluted blood with lysis buffer), 3.3, 0.33; to 0.03%. Similarly, serum samples were serially diluted in PBS in a range from 100% (undiluted serum), 10%, 1% and 0.1%.

Both serially-diluted serum and blood lysate samples were used as sample matrices for μPAD sensor testing. Each dilution was spiked to a final concentration of 133 nM with r*Pf*LDH before 30 µl samples of each were introduced to the μPAD sensor and tested for captured r*Pf*LDH as detailed in Sect. 2.2.3–2.2.5 above. In the case of blood lysate, a further modification was applied: after aptamer-target exposure, the paper sensor used for blood lysate analysis was washed 3 times with PBS-T containing 1% ^v^/_v_ hydrogen peroxide to decrease the red colour imparted by blood [20].

### Statistical analysis

For each presented sample, three separate µPADs were fabricated and tested and are presented below as univariate plots [34]. Presented results in text represent the means ± standard errors of the means.

Statistical tests and fitting of models was performed using RStudio v.1.2.5033, operating R v3.6. For all statistical tests, the level of significance, α, was set at 0.05. Significant difference in the means of datasets comprising more than two samples was tested by one-way analysis of variance (ANOVA), using Tukey’s HSD post hoc test to identify samples significantly different from their counterparts. Results of ANOVA i.e. calculated F-statistics, are reported in the form of annotation to every graph where significant differences between samples are discussed.

Comparisons of the means of two-sample datasets were conducted using two-sample, two-tailed *t* tests. Testing of one-sample datasets i.e. contrast data was conducted using one-sample *t* tests, with the null hypothesis that the means of the sample = 0.

Similar to previous studies [12, 35] the kinetic parameters of the µPAD sensor’s affinity to *rPf*LDH concentrations were fitted via nonlinear least-squares fitting to Langmuir binding isotherms (Eq. 3).

## Results

The APTEC-based µPADs were constructed as depicted in Fig. 1 and detailed in Sect. 2.4. Preliminary analysis indicated that the optimum time for 30 µl samples to travel the length of the sensor was 10 min (Additional file 1: Fig. S1): this time between the addition of samples, wash buffers and reagents was the same across all sensors.

The ability of all of the aptamer sequences tested in this study (Table 1: LDHp11, rLDH4, rLDH7, pL1 and 2008s) to detect r*Pf*LDH in the µPAD diagnostic format was initially screened. For each individual sensor, the contrast evident for each sensor’s test zones (compared to the control zones) were additionally calculated using Eq. 2 and graphed for all subsequent analyses (e.g. Figure 2C). Experimental conditions producing significant contrasts across multiple sensors were identified using 1-sample *t* tests.

Figure 2A shows colour-enhanced images, comparing the colorimetric responses of sensors constructed using the different tested aptamer sequences (the original images as-captured are presented in Additional file 1 Materials, S2). The analysed, background-corrected, colorimetric intensities of the test zones (Zones 1 and 3) of each µPAD (*∆I*) are presented in Fig. 2B and compared to the colour intensities measured at the control zone (Zone 2).

By itself, the *rPf*LDH biomarker showed little nonspecific binding to the BSA-blocked sensor surface. This is seen by the very slight purple colour evident in the other areas of the sensor (compared to the background) other than the test zones (Fig. 2A). Some dispersion of the purple colour outside the printed confines of the sensor is evident in some of the sensors (e.g. rLDH4 in Fig. 2A). The formation of colour outside the printed sensor is attributed to the low height of the wax layer after annealing, resulting in the transfer of liquid droplets outside the sensor onto the unmodified paper’s surface. Lacking a BSA block, the subsequent nonspecific attachment of r*Pf*LDH proteins to the surface would result in a purple colour forming after the application of Malstat reagent.

**Fig. 3:**
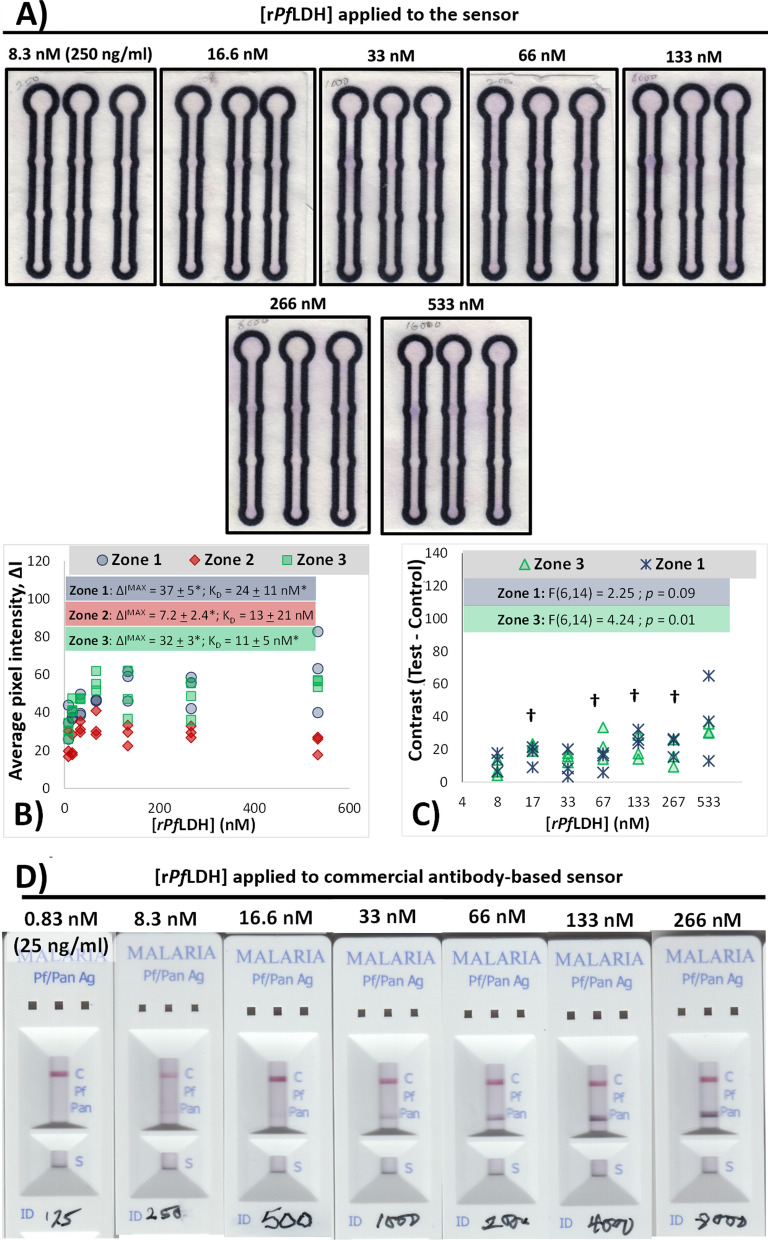
Responses of rLDH7-based µPAD APTEC sensors towards varying concentrations of r*Pf*LDH target. µPAD sensors were constructed using the rLDH7 aptamer and exposed to varying concentrations of r*Pf*LDH before the attached r*Pf*LDH was visualised using the Malstat colour assay. **A** Contrast-enhanced photographs of µPAD sensors following application of varying concentrations of r*Pf*LDH and subsequent colour development. Annotations indicate the concentration of rPfLDH in the sample applied to the sensor. The original scanned images are presented in Additional file information (Additional file 1: Fig. S3) and were used to construct the measurements of *∆I* presented in Fig. 3B and C. **B** Scatterplot of the measured colour intensities at the various zones of the µPAD as a function of the applied r*Pf*LDH concentration. Binding affinity curves based on concentration-dependent detection fitted from colour intensity using Eq. 3 are presented as lines, with measurements reporting the mean ± standard error of the estimated variable. The kinetic parameters estimated from the fit are annotated for each zone. *—indicates that a particular fitted variable. Annotated has p < 0.05 for the null hypothesis of this value being = 0 (determined via t statistic testing). A baseline ${I}_{0}$ of 22 pixels was set for all fitted models, based on the average Zone 2 responses. **C** Semi-logarithmic scatterplot of the measured contrast between test and control zones. †—indicates that the values for Zone 1 contrast have mean values that are significantly above zero; (*p* ≤ 0.05; 1-sample *t*-test). **D** Responses of commercially-available, antibody-based, RDT devices (*OnSite*^®^ Malaria Pf/Pan Ag Rapid Test) towards varying concentrations of *rPf*LDH target used in this study. The target protein was dissolved in buffer, before being applied to the RDT. Images were captured and contrast-enhanced, as was performed for APTEC tests. The “Pan” test line is specific to *P*LDH, while the “Pf” test line is specific to PfHRP2

During aptamer screening, the BSA-blocked control zones (Zone 2) of the majority of μPAD sensors produced little visible colour after addition of NBT/PES (Fig. 2A), even in sensors where diffusion of the target across the sensor to Zone 3 is evident (e.g. sensors comprising 2008s and rLDH7, Fig. 2A). This resulted in the low colorimetric intensities (*∆I* values) measured for Zone 2 samples across the screened aptamers: an average *∆I* response of 17.6 ± 7.1 pixels for the control zones in sensors comprising LDHP11, pL1, 2008s and rLDH4 was found (Fig. 2B). This – together with the lack of evident colour outside of zones 1 and 3 for the screened aptamers – indicates that little to no rLDH enzyme nonspecifically attached to the paper sensor surfaces. The sole exception to this was a single test µPAD for rLDH7 (Fig. 3B), which elevated both the average and the variance of colorimetric responses in zone 2 for this sample, increasing it to 22.6 ± 20.29. This is attributed to accidental overflow of the EDC/NHS solution during activation and subsequent immobilization of the streptavidin and biotinylated aptamer during the wash steps. Despite this single reading, no significant differences in the means of the control zones was evident across the tested aptamers (F(4, 10) = 1.28; *p* = 0.36).

Only two of the screened aptamers produced sensors that exhibited strong colour production at the test zones (Zones 1 and Zone 3): 2008s and rLDH7 (Fig. 2A). Analysed as a group, (Fig. 2B), the use of 2008s produced statistically-significant colorimetric signal in Zone 1 (*∆I* = 70.6 ± 4.3 pixels), compared to Zone 2 (*∆I* = 21.3 ± 10.7 pixels) via Student’s *t*-tests (* annotation). This indicated the ability of the immobilized 2008s aptamer to successfully capture r*Pf*LDH at the Zones 1 and 3 (Fig. 2B and C).

Significant sensor variation was evident within this study, resulting in a wide dispersion of *∆I* values within the sensors and preventing ready comparison of the sequences by examination of group responses presented in Fig. 2B. To normalize differences in the colour development between sensors of the same composition, the contrast between the test zones and the control zones for individual sensors was also determined, subtracting the *∆I* values of the test zones from the control zones (Fig. 2C). Evaluated by contrast measurements, Zone 1 regions of both 2008s-based sensors and rLDH7-comprising sensors exhibited statistically-significant contrasts (via one-sample *t* tests, **‡** annotation) that are similar to one-another in magnitude.

Due to the strong signal generated by the rLDH7 aptamer, and to compare this sensor’s function with the findings from Frith et al. [12], further µPAD investigations were conducted using this aptamer alone, and the specificity, sensitivity, as well as performance of the sensor in blood samples, were tested.

The influence of *rPfLDH* concentration on the intensity of the colorimetric signal was evaluated. Independent sensors were fabricated and each exposed to a single concentration of the *rPfLDH* biomarker. Figure 3A presents contrast-enhanced images of the colorimetric response of the µPAD APTEC diagnostic test in the presence of varying concentrations of r*Pf*LDH applied as a sample. From data extracted from these images, Fig. 3B graphs the dependency of the measured intensities of the colour on protein concentration, while Fig. 3C presents a semi-logarithmic plot of the sensor constrast, to demonstrate the limits of detection by the μPAD sensor. An increase in the intensity of colour within the test zones with increasing concentrations of r*Pf*LDH (Fig. 3A), indicates a concentration-dependent capture of the target by the immobilized aptamers in Zones 1 and 3.

A slight increase in purple colour throughout the sensor is also evident with increasing r*Pf*LDH concentration (e.g. comparing 8.3 nM and 66 nM samples in Fig. 3A), indicating nonspecific attachment of the target enzyme to the paper surface during its diffusion along the sensor. This is represented during colorimetric analysis by the concentration-dependent increase in the measured colour intensities in Zone 2 of the sensor (Fig. 3B).

Despite the above, successfully concentration of the enzyme-derived colour signal by the immobilized aptamers was observed. Comparison of the dependence of colorimetric sensor responses on the concentration of the target (Fig. 3B) indicated that significantly-larger amounts of r*Pf*LDH bound to the test zones (Zones 1 and 3) compared to the nonspecific binding observable at Zone 2. Through contrast analysis, the lowest concentration of r*Pf*LDH capable of creating statistically-significant contrast between the test and control zones for individual sensors was determined to be 16.6 nM († annotations in Fig. 3C). At higher concentrations of target, the sensor produced significantly more colour in Zone 1 compared to Zone 2 more-or-less consistently. Some tested concentrations did not produce sufficient contrast, due to a combination of high nonspecific signal (in the case of sensors exposed to 33 nM of r*Pf*LDH in Fig. 3A) and/or high inter-sensor variation (in the case of sensors exposed to samples containing 533 nM of r*Pf*LDH, despite the evident signal in visible in Fig. 3A).

The sensitivity of the APTEC sensor was compared to that of a commercially-available, antibody-based test, exposed to a range of r*Pf*LDH (Fig. 3D). The test line labelled “Pan” is specific to *P*LDH; the visibility of this test line increased with increasing concentrations of applied r*Pf*LDH. Similar to the results obtained with the APTEC sensors (Fig. 3A), a faint test line appears at target concentrations between 8.3 nM to 16.6 nM, with a stronger signal developing thereafter. Given the faintness of the test line visible at 8.3 nM, the empirical limit of detection for the r*Pf*LDH with these RDTs was set to this concentration.

The ability of the immobilized rLDH7 aptamer to specifically detect the presence of the target protein in a complex matrix such as serum and blood lysate was also investigated. Spiking constant concentrations of r*Pf*LDH protein into varying concentrations of serum was selected as a means of determining the effect of serum concentration on the performance of the APTEC diagnostic device.

**Fig. 4:**
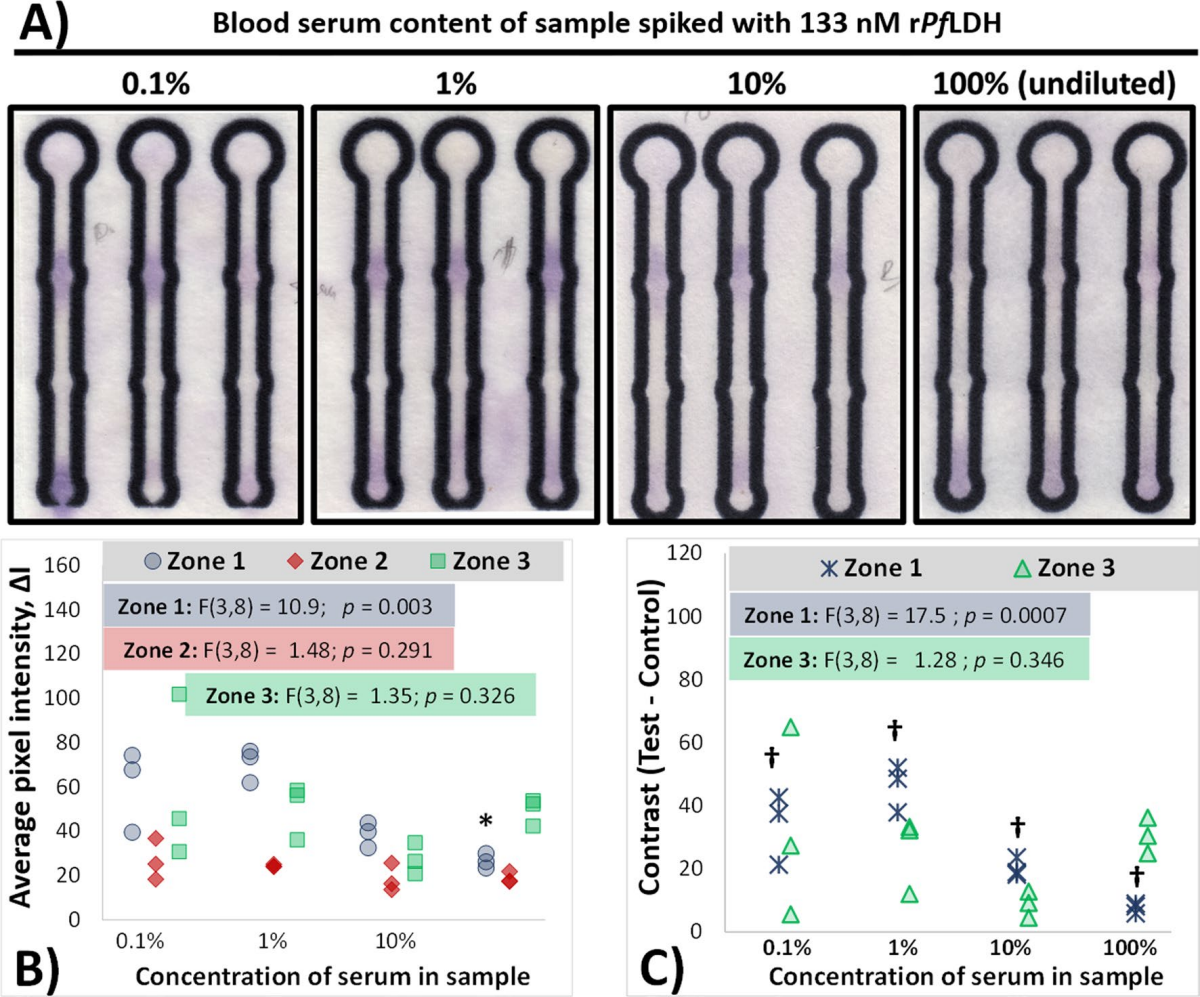
Effect of serum concentration on the ability of the rLDH7-based APTEC µPAD platform to detect the presence of 133 nM r*Pf*LDH spiked into the sample. **A** Colour-enhanced photographs of the colorimetric response of the rLDH7 µPAD in the presence of 133 nM r*Pf*LDH prepared in varying concentrations of serum. **B** Scatterplot comparing the influence of serum concentration on resultant colour intensities in the sensors presented in (**A**). ANOVA results are presented as annotations inset within the graph. *- indicates serum concentrations that resulted in Zone 1 colorimetric responses significantly lower to those found in 0.1% serum samples (*p* < 0.05; Tukey’s post hoc test). **C** Scatterplot comparing the contrast (ΔI of test zones – ΔI of control zone 2) for individual sensors. ANOVA results are presented as annotations inset within the graph. **†**—indicates *p* ≤ 0.05 for a 1-sample *t* test for Zone 1 average contrasts, testing againt the null hypothesis that the average = 0

Figure 4 presents the effect of increasing serum content in the sample matrix on subsequent μPAD sensor colour development, using a fixed concentration of r*Pf*LDH throughout (133 nM).

Despite a lack of strong colour contributed by the serum sample, higher concentrations of serum significantly decreased μPAD sensor responses. Relative to the background signal, a significant decrease in the intensity of Zone 1 colorimetric intensity is evident with increasing serum concentration: from ∆*I* values of 60.5 ± 10.6 at samples containing 0.1% serum to 26.6 ± 1.88 in undiluted serum samples spiked with r*Pf*LDH (Fig. 4B, * annotation). A similar decrease in signal intensity was noted when 133 nM of r*PfL*DH was measured using the antibody-based RDT in the presence of undiluted serum, when comparing the intensity of the test line to the same concentration of r*PfL*DH dissolved in buffer (Additional file 1: Fig. S3).

**Fig. 5:**
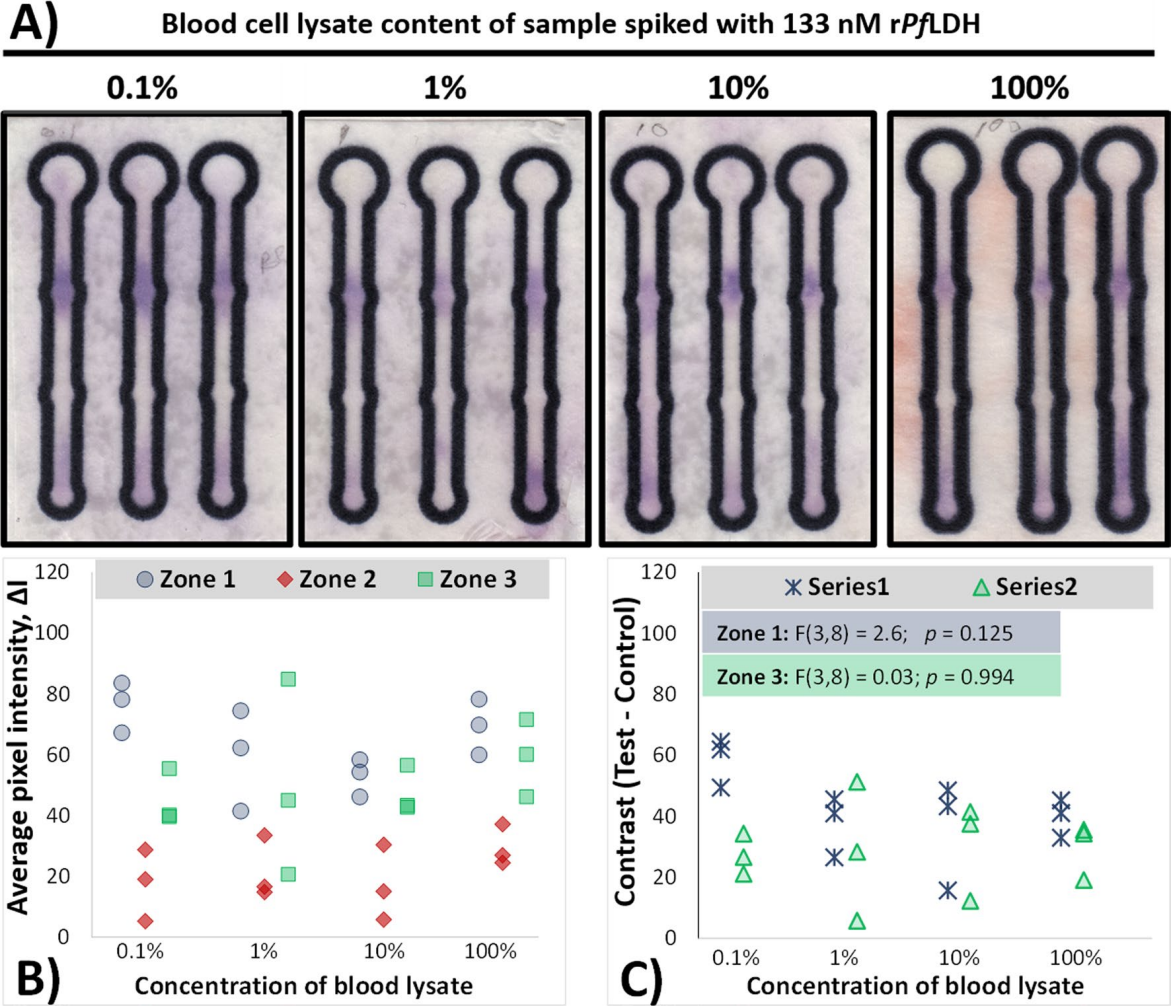
Effect of blood lysate concentration on the ability of the rLDH7-based APTEC µPAD platform to detect the presence of 133 nM rPfLDH spiked into the sample. **A** Photographs of the colorimetric response of rLDH7 µPADs in the presence of 133 nM r*Pf*LDH in varying concentrations of blood lysate. **B** Comparison of colour intensities produced by the tests presented in (**A**). **C** Scatterplot comparing the contrast (ΔI of test zones – ΔI of control zone 2) for individual sensors. ANOVA results are presented as annotations inset within the graph

A similar study to the above was applied to blood lysate spiked with r*Pf*LDH. Figure 5 displays the influence of the µPAD APTEC biosensor’s response to the presence of r*Pf*LDH in varying concentrations of blood lysate.

Little to no influence of blood lysate content on the intensity of developed colour in Zone 1 was noted.

(Fig. 5), indicating that the µPAD device could detect r*Pf*LDH in blood matrices. Consistent with previous studies, colour change in Zone 1 indicated biorecognition of the rPfLDH by immobilized aptamer (Fig. 5A) in blood. Unlike the decreases in colour development with increased serum concentration in the sample (Fig. 4), a relatively consistent colour development in Zone 1 is evident across the tested blood lysate concentrations (Fig. 5A). This resulted in consistent measured colorimetric intensities (*∆I* = 64.6 ± 13.18 across all sensors; Fig. 5B) and contrast values (the lack of a statistically-significant ANOVA for Zone 1 inset in Fig. 5C). A similar, slight lowering of sensor response was also observed when monitoring r*Pf*LDH in undiluted blood lysate using the commercial RDT (Additional file 1: Fig. S3).

## Discussion

µPADs are designed with a variety of configuration with varying test and control zones for either multiplex (e.g. [36]); or single-target detection [22]. For the purposes of this study, this µPAD configuration lacked a positive control zone in favour of two separate test zones at either ends of the sensor path (Zones 1 and 3, Fig. 1). The aptamers immobilized at Zone 1 were intended to capture a significant amount of the r*Pf*LDH, while residual capture of r*Pf*LDH occurred at Zone 3, as the samples were removed from the sensor by wicking action. The control zone was positioned between these two test zones, to ensure that any colour formed was not due to diffusional constraints of the enzyme across the sensor (which may result in its accumulation around Zone 1) or its accumulation at the end of the sensor strip during wicking (which may accumulate enzyme near the third zone). For this reason, the differences in colour intensities between Zones 1 and 2 for sensors are discussed as indicative of sensor function, while colour formation in Zone 3 was taken as evidence of diffusion of rPfLDH across the length of the sensor’s surface and was used as confirmation that Zones 1 and 2 were adequately exposed to r*Pf*LDH.

The μPAD design has some features which make the test attractive. The current format – with two separate test zones – confirms the distribution of reagents across the entire test area and allows comparison between the signal at each zone to take place. The general decrease in signal intensity between zones one and zones three in most of the functioning aptasensors in the above studies demonstrates successful aptamer-mediated capture of the target at Zone 1. The µPAD has two separate steps to ensure signal specificity. The first is the capture of *P*LDH by the aptamer and the second is the specificity of the enzyme assay employing APAD as a unique *Pf*LDH substrate. This is an advantage over previous aptamer-based approaches to detect *P*LDH [12] or *Pf*HRPII [4] which use only one step to ensure specificity. The enzyme-catalysed reaction has the further advantage of signal amplification.

The successful capture of *Pf*LDH by 2008s (Fig. 2) was anticipated by previous reports [2, 18]. When immobilized via streptavidin on a microtitre plate, this aptamer was demonstrated to specifically capture *Pf*LDH for subsequent colorimetric detection via the Malstat assay format. The work in this current study, however, demonstrates the utility of translating this assay format into a low-cost paper format – rather than a microplate assay. pL1 did not exhibit any significant colour development when immobilized onto paper, in contrast to expected results [2] with similar attachment to streptavidin-modified surfaces. Such differences in aptamer affinity for the target are however expected, since affinity of aptamers for their targets are frequently dependent on the nature of the substrate, method of immobilization and buffering/ sample conditions [37].

Affinity analysis (presented in Fig. 3B) confirmed specific colour development occurring in the aptamer-modified zones of the μPAD sensor. This is indicated by the much higher maximal colour intensities at the test zones for Zones 1 and 3 respectively, compared to the nonspecific binding measured at Zone 2 (*ΔI*^*max*^ values inset in Fig. 3B). Furthermore, both Zone 1 and Zone 3 produced concentration-dependent responses that were reasonably-fitted by the Langmuir model employed (* annotations for both *ΔI*^*max*^ and K’, insets of Fig. 3B) and similar maximal colour intensities. The fitted K’ (24 ± 11 nM for Zone 1 responses) are reasonably close to two similar APTEC studies in general and to rLDH7’s reported binding affinity. Specifically, Frith et al. [12] reported ~ 40 nM as the *K*_*D*_ for the binding interaction between rLDH7 aptamer and r*Pf*LDH using microplate ELONAs. APTEC-based r*Pf*LDH sensors using other aptamers reported *K*_*D*_ values of 6.2 and 43 nM for pL1 and 2008s aptamers, respectively, albeit using microplate platforms to determine colour development [2].

The empirical limit of detection for r*Pf*LDH using the APTEC sensors in this work (16.6 nM) is considerably higher than those reported using other forms of Malstat-based PLDH capture assays, e.g. Markwalter et al. [17] reported a 25.7 pM limit of detection when using anti-*Pf*LDH antibody-coated magnetic beads. However, it compared favourably with the limit of detection found in this study using a commercial antibody-based RDT (8.3 nM). This particular brand of RDT has been previously found to provide satisfactory diagnosis of malarial parasitaemia, compared to microscopy and PCR-based detection [38, 39]. The detection of the recombinant target r*Pf*LDH by this RDT indicated that the relevant epitopes recognized by the RDT test are adequately presented by the target molecule in order to be recognized by the antibodies within the RDT. This—together with the enzyme activity present in the target to generate signal via APTEC sensors—provides some validation that the structure of the r*Pf*LDH molecule resembles that of natively-expressed *Pf*LDH,

The decrease in colorimetric sensor intensity with increased serum protein content (Fig. 4) indicated some interference in r*Pf*LDH capture by the immobilized rLDH7 at higher concentrations of serum proteins, and appeared to affect the performance of both the APTEC sensor reported in this study and the commercial RDT (Additional file 1: Fig. S3 and Additional file 2). Despite this, the detection of r*Pf*LDH, relative to the control zones, was possible for all tested serum dilutions.

Spiking blood lysates with proteins is a useful method to reproduce the natural environment (blood) of parasite derived material and has been used to evaluate other plasmodial biomarkers [40]. In the presence of blood lysate (Fig. 5), a similar, albeit slighter, decrease in signal intensity with increasing blood lysate concentrations is evident. Unlike the study conducted in serum dilutions (Fig. 4), the influence of blood lysate concentration on contrast was not statistically-significant (Fig. 5C). Several factors may have been involved. Firstly, a comparison of the Zone 2 responses (Fig. 5A and B) shows some colour contribution to the sensor by the blood lysate at higher concentrations, which may affect the perception of colour on the sensor after Malstat assaying. Secondly, similarly to the blood serum study in Fig. 4, increasing concentrations of blood lysate slightly decreased Zone 1 responses (Figs. 5A and 5B), indicating that blood lysate components may inhibit binding of the r*Pf*LDH to the test zones. Preparation of the red blood lysate diluted the blood sample threefold, in turn diluting the inhibiting components in this sample. Other APTEC reports showed similar effects [2], with 2008s-based APTEC assays successfully detecting the presence of *Pf*LDH in clinical blood samples of infected patients.

All tested concentrations of blood lysate generated a visible purple colour at Zone 1, as well as significant contrast values (Fig. 5C, **†** annotation). Blood lysate content therefore has little effect on the colourimetric intensity (Fig. 5), indicating that the µPAD device could successfully detect r*Pf*LDH in blood samples.

## Conclusion

Wax-printed channels on paper were fabricated and used to selectively attach r*Pf*LDH-binding aptamers at specific sites to create a proof-of-concept microfluidic paper-based analytical device (μPAD). This configuration was used to screen several r*Pf*LDH-binding aptamers (aptamers LDHp11, rLDH4, rLDH7, pL1 and 2008) for their ability to selectively capture r*PF*LDH and to subsequently report on their successful interaction using the Malstat colorimetric assay. Of the aptamers investigated for the construction of the µPAD, rLDH7 was selected for further characterization, and tested for its analytical performance in serum and blood samples. While rLDH7 was selected as a capture aptamer – on the basis of its relative lack of characterization in the literature – 2008s was also noted to produce a significant colorimetric response during screening. This, combined with reports of its preferential capture of LDH from *P. falciparum* over other species of malaria (notably *P. vivax*) during APTEC in in other reports [2] makes the 2008s aptamer an attractive sequence for future investigation in μPAD configuration.

A limit of detection in the nM region was noted for the proof-of-concept, aptamer-based, rLDH7 μPAD sensor described in this study, which approached that of commercially-available antibody-based RDTs. Further scope exists in optimizing the design of these sensors: improving the loading of streptavidin at test zones to increase aptamer attachment and subsequent enzyme retention and further standardizing the EDC/NHS functionalization of the cellulose surfaces to concentrate the signal into smaller zones to improve sensitivity are currently being researched.

The retention of significant colour development in the presence of undiluted blood serum and blood lysates indicates that this particular µPAD configuration may thus offer a means of malaria diagnosis purposes within these matrices in the future. While the recombinant r*Pf*LDH protein appeared to function well as a target – both providing detectable LDH enzyme activity by Malstat as well as presenting suitable epitopes for antibody-based detection by the RDT – additional studies examining native *Pf*LDH in cultured malaria parasite lysates and, ultimately, infected patient blood samples should be undertaken to validate the sensor’s functionality towards diagnostic field samples.

## Supplementary Information

**Additional file 1**: **Figure S1.** Time-dependent distance travelled by 30 μl of 133 nM rPfLDH in the μPAD. **Figure S2.** Original digitally-captured images of μPAD sensors, before digital processing. **Figure S3.** Contrast-enhanced images, comparing responses of commercial RDTs exposed to rPfLDH dissolved in undiluted blood serum and blood cell lysate samples.

**Additional file 2.** Spreadsheet summarising the numerical data obtained from ImageJ analysis for each μPAD sensor response and associated statistical tests used in generating  and analysing results presented in this manuscript.

## Data Availability

All data generated or analysed during this study are included in this published article. Unedited images of the µPAD sensors are in the Additional file Materials (Additional file 1: Fig. S2). The numerical data extracted via ImageJ, descriptive and inferential statistics relevant to each study in this article are attached as a Additional file to the published article.
