# Supplementary material for: A microfluidic paper analytical device using capture aptamers for the detection of PfLDH in blood matrices
Source: Malar J. 2022 Jun 7;21:174. doi: 10.1186/s12936-022-04187-6 (PMC9172074; doi:10.1186/s12936-022-04187-6)
Supplement: Supplementary file 1 — Additional file 1: Figure S1. Time-dependent distance travelled by 30 μl of 133 nM rPfLDH in the μPAD. Figure S2. Original digitally-captured images of μPAD sensors, before digital processing. Figure S3. Contrast-enhanced images, comparing responses of commercial RDTs exposed to rPfLDH dissolved in undiluted blood serum and blood cell lysate samples. [file 12936_2022_4187_MOESM1_ESM.pdf]

Supplementary information:

A: Determination of sample migration time during sensor operation

To determine the average time taken for samples to migrate along the sensor surface, the time-dependent wetting caused by an aliquot of r*Pf*LDH applied to a dry sensor was evaluated. Fig. S1A and 1B describes the distance travelled by a volume of 30 µl r*Pf*LDH (133 nM) as a function of time.

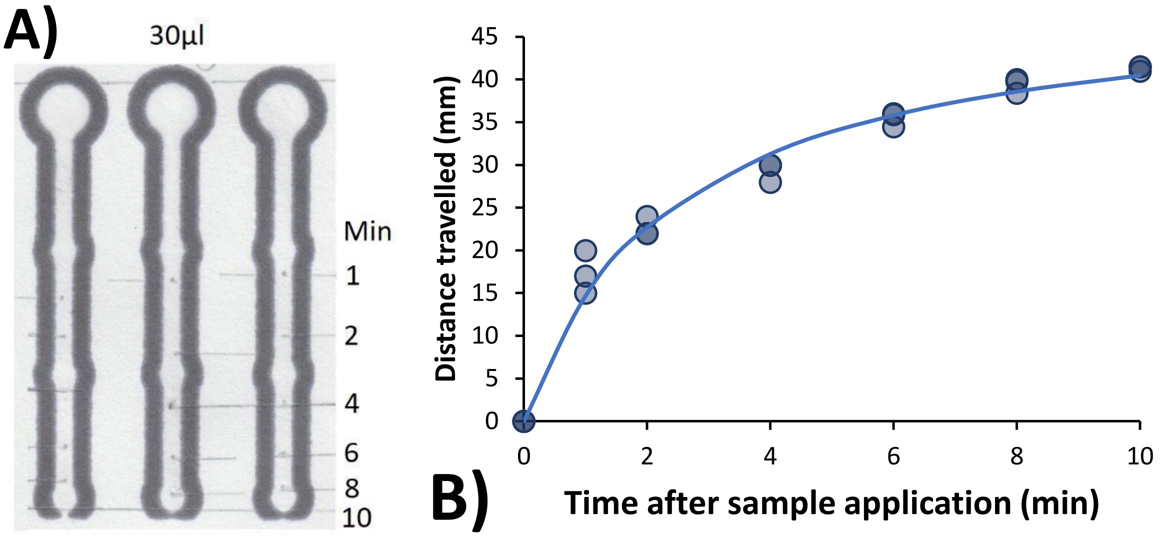

Fig. S1: Time-dependent distance travelled by 30 µl of 133 nM r*Pf*LDH in the µPAD.

A) Photographs of distances travelled by 30 µl of r*Pf*LDH applied to the sample zone at different times (1, 2.5, 5, 10, 15, 20, 30 min).

B) Scatterplot of the measured travelled distances as a function of time. The time required for 30 µl of 133 nM *Pf*LDH to cover 42 mm (the entire length of the printed µPAD) was recorded. The experimental measurements here were fitted using equation 5 (line) which determined *B* to be 1.88×10^{-3}.

Fig. S1A shows the photographs of the record of the dynamic capillary mobility of 30 µl of 133 nM r*Pf*LDH applied to the sample area. The distance travelled by the sample was estimated by measuring the leading edge of the wetting of the paper. A hyperbolic dependence of the distance travelled by the sample vs. time is evident from these, with successive measurements showing smaller increments of distance. As indicated in Fig. S1B, 30 µl of r*Pf*LDH could travel the whole length (~50 mm) of the µPAD within 10 minutes.

Fig. S1B graphs the distance travelled as a function of the time after sample application, fitted to a previously-reported time-dependent model for fluid flow in porous materials with multiple pore sizes [41]. A similar dependence between this study and the reported study occurred, with distance travelled being related to the square root of travel time ($l = B\sqrt{t}$). From this graph, the time constant K_t, which signifies the limit of the linear region of the time-distance dependence, was estimated to be 2.29 min. After the 2.29 min, the capillary action of the fluid front continued at a slower pace compared to the initial turbulent flow.

As the flow profile of r*Pf*LDH solution is expected to be dependent on the volume of solution applied to the sample area as well as the length of time of flow, similar to prior reports for porous media [25,42], the application volume was thereafter standardised at 30 µl. An exchange of the media throughout the sensor after the application of 30 µl aliquots was, correspondingly, expected to occur within a minimum time of 10 minutes throughout the study.

B: Unprocessed images of the μPAD sensor responses reported in the study

The as-captured images of the APTEC μPADs after application of Malstat reagent are presented below in Fig. S2.

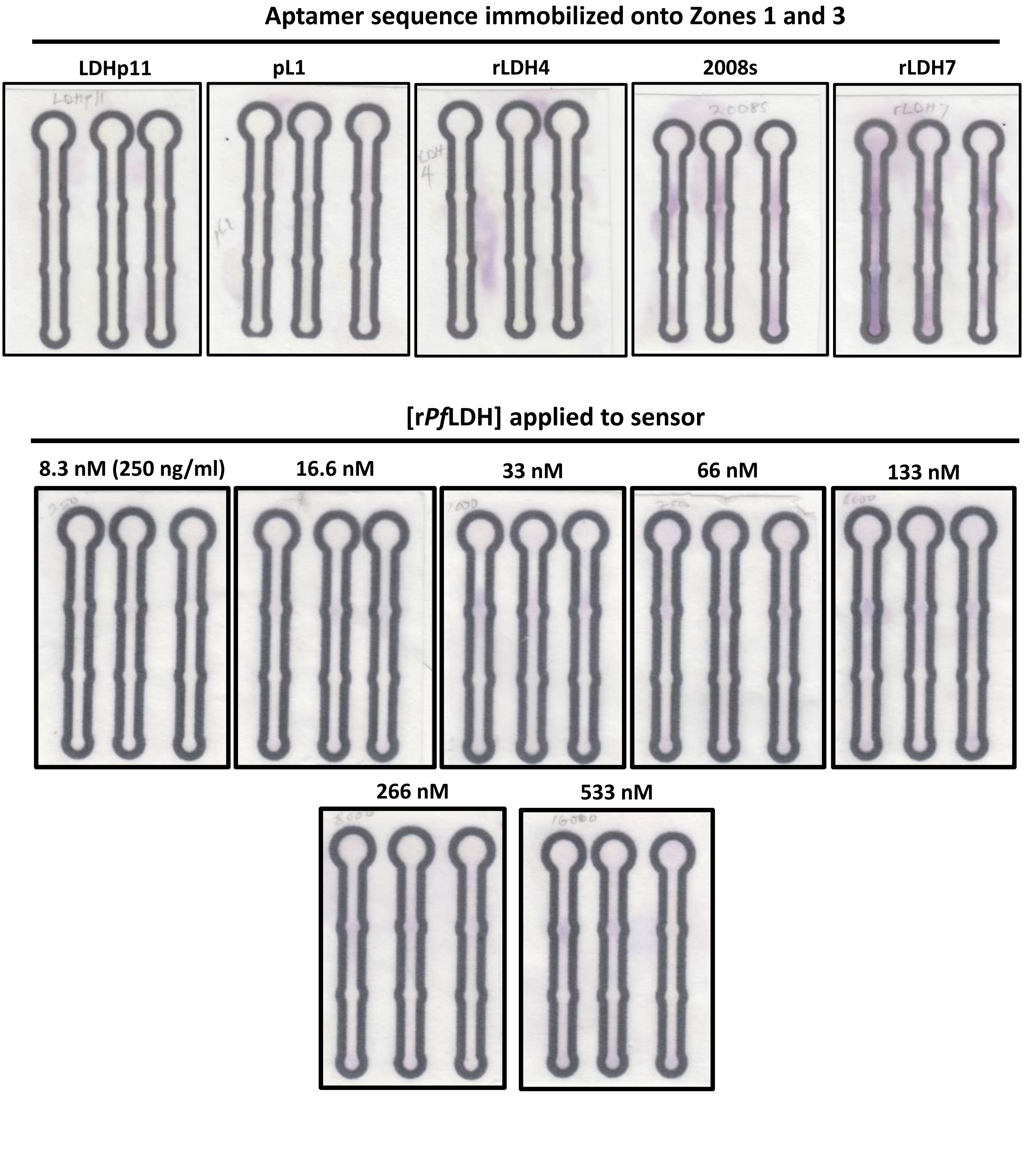

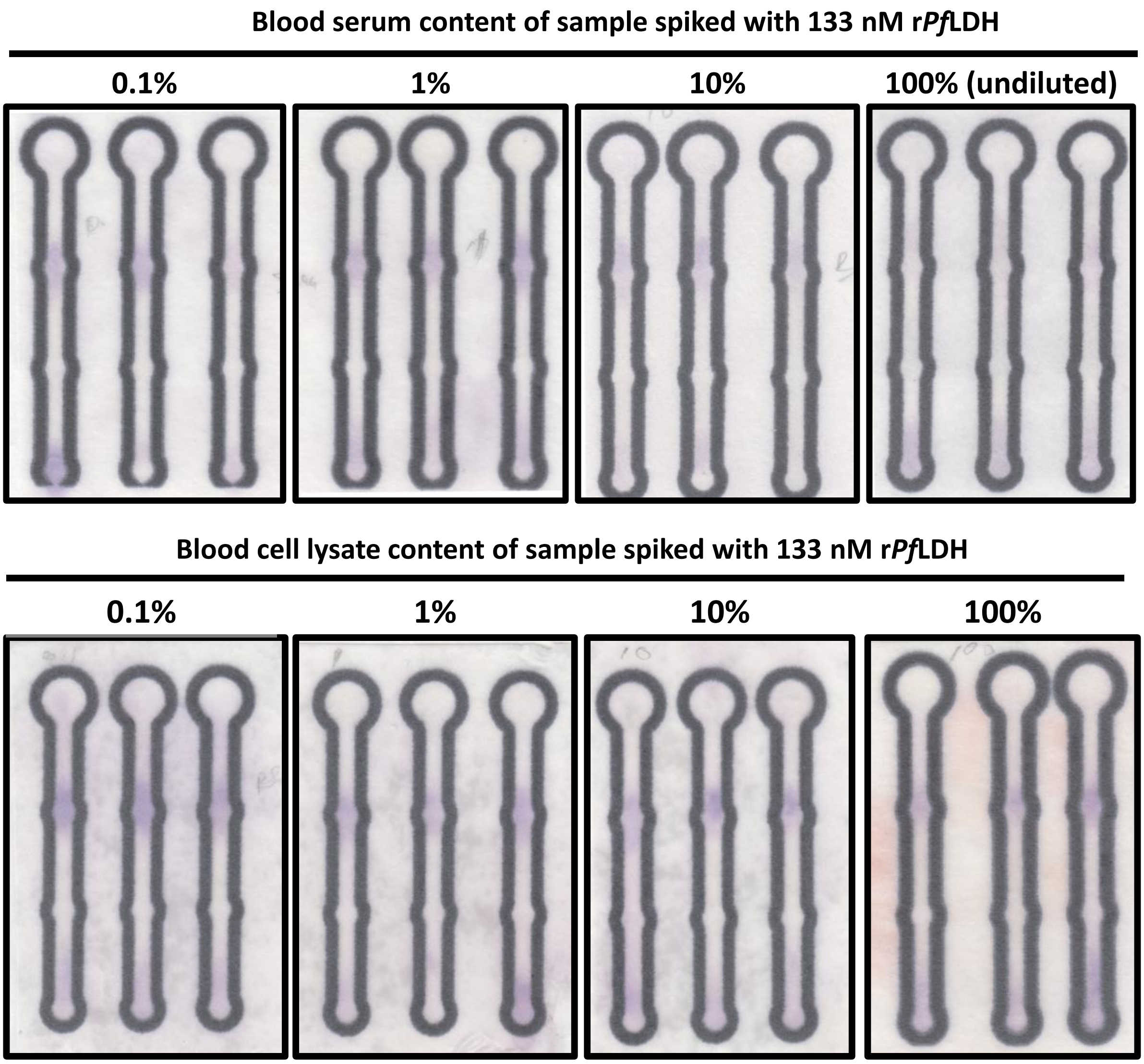

Fig. S2: Original digitally-captured images of μPAD sensors, before digital processing.

C: Responses of antibody-based RDTs to r*Pf*LDH dissolved in serum and whole blood

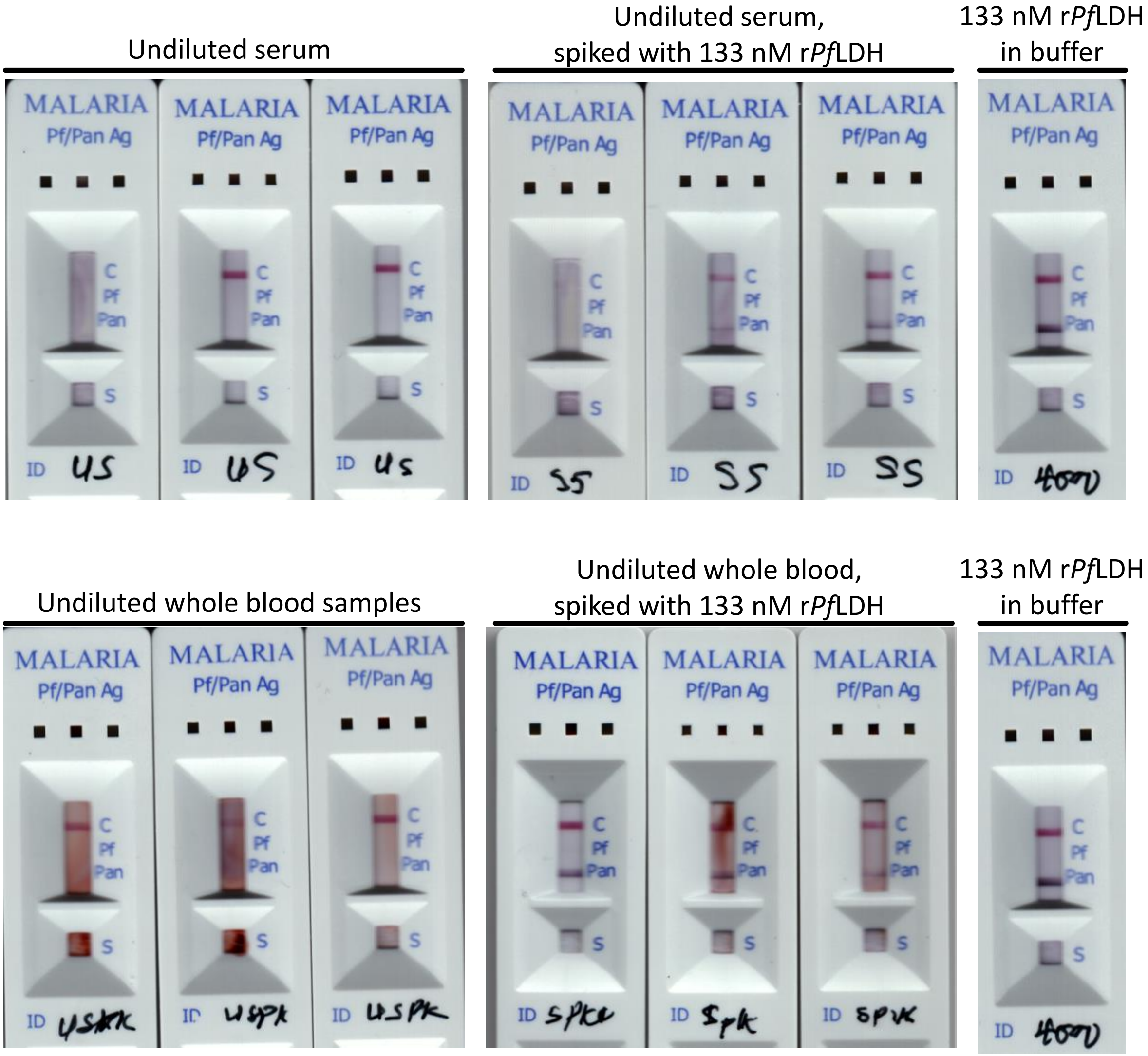

Fig. S3: Contrast-enhanced images, comparing responses of commercial RDTs exposed to r*Pf*LDH dissolved in undiluted blood serum and blood cell lysate samples.

Target protein was dissolved to 133 nM in the serum or blood cell lysate matrices before being applied to the sensor. For comparative purposes, the responses of an RDT test to the same concentration of r*Pf*LDH in buffer (extracted from Fig. 4D of the manuscript) is also presented. *n* = 3 independent measurements were conducted for each sample and are presented**.**

Whole blood samples were used rather than the blood lysate used for APTEC devices, as manufacturer guidelines did not advise the use of blood lysate.
